# The roles, responsibilities and perceptions of community health workers and ward-based primary health care outreach teams (WBPHCOTs) in South Africa: a scoping review protocol

**DOI:** 10.1186/s13643-019-1114-5

**Published:** 2019-08-05

**Authors:** Euphemia Mbali Mhlongo, Elizabeth Lutge

**Affiliations:** 1 Discipline of Nursing, School of Nursing and Public Health, University of KwaZulu-Natal, Private Bag X54001, Durban, 4000 South Africa; 2 KwaZulu-Natal Department of Health, Pietermaritzburg, South Africa

**Keywords:** Community health worker, Primary health care, Ward-based primary health care outreach teams

## Abstract

**Background:**

Human resource for health (HRH) challenges jeopardise the South African health system, undermining the efforts made to curb the burden of disease. There is a demand for a category of health workers, which will meet the basic health needs of people at the grassroots level to ensure accessible, affordable health care using appropriate technologies acceptable to the recipients of care. The ward-based primary health care outreach teams are well placed to provide community-based primary health care services, which encompass activities in communities, households and referral networks with community-based providers. This study aims to elicit factors enabling or undermining the effectiveness of ward-based primary health care outreach teams in KwaZulu-Natal.

**Methods:**

The search strategy of this scoping review will be guided by Arksey and O’Malley’s scoping review methodology framework. The following electronic databases will be searched: PubMed, Google Scholar, Science Direct, Clinical key and from EBSCOhost platform and Dissertation via World Cat. The selection of study will involve three stages of screening. The principal author will conduct the title screen of articles from the databases and remove the duplicates. Two authors will independently conduct the abstract and full text screening, and articles that meet the eligibility criteria will be included for the study. Data will be extracted from the studies included, and the emerging themes will be analysed using NVIVO software. A quality assessment of the included studies will be determined through a mixed method appraisal tool (MMAT) version 2011.

**Discussion:**

Ward-based primary health care outreach team (WBPHCOT) evidence, acceptability, preferences or practice effectiveness studies will be identified. Further expected results also include identification of knowledge gaps in primary health care practice as well as inform future research required. Findings will be disseminated electronically, in print and through peer presentation, conferences and congresses.

Results from this scoping review will be useful to inform local and the South Africa National Health Insurance programme managers concerning the impact ward-based primary health care outreach teams have on the national health care system and on the health of the population.

## Background

Community health workers had their origins in China in the 1920s and were precursors to the “barefoot doctor” programme which was a movement in the 1950s [1–3]. Community health workers have been defined as “a diverse group of health workers whose common characteristic is their work outside of health facilities directly with people in their homes, neighbourhoods, communities and other non-clinical spaces where health and disease are produced”. In the 1960s, community health worker programmes emerged in Indonesia, India, Tanzania and Venezuela [3].

Just over a decade later, an international conference on primary health care (PHC) was held at Alma Ata where a Declaration of Health for All by the year 2000 was made. The Alma Ata Declaration of 1978 articulated the urgent need for all governments, health and development workers and the communities, to protect and promote the health of all the people of the world [4]. Primary health care was identified as the key intervention in attaining the Alma Ata Declaration. The community health workers’ role in providing PHC was highlighted in the Declaration [5]. These categories of staff need to be suitably trained to work as a health team in order to respond to the identified and expressed health needs of the community. Community health worker programmes in low-, middle- and high-income countries proved to be successful in the 1980s in countries like Brazil (community health agents), Bangladesh (family welfare assistants) and Nepal (female community health volunteer programme) [3].

During the 1990s, the World Health Organization promoted task-shifting to counteract the overstretched health care system [6]. Pakistan launched their large-scale, community-based lady health worker programme in 1992, followed by Ugandan community health worker (CHW) programme (village health teams) [7, 8]. In 2004, Ethiopia embarked on a training programme of their health extension workers [3, 9], while in 2005, India introduced her CHW programme which is now the largest in the world (accredited social health activists) [3]. Although CHWs have different names in different countries (home-based carers, community caregiver), their roles overlap. For example, in low-income countries, CHWs are instrumental in reducing childhood malnutrition, improving maternal and child health, increasing access to family planning and controlling the spread of HIV, malaria and TB infections [3].

In middle-income countries, CHWs form important health team members and are critical for PHC provision including health promotion [8, 10, 11]. Therefore, this study review will include research articles from low and middle incomes with South Africa in mind. Evidence shows that in the USA, CHWs can contribute to the management of hypertension, diabetes and HIV and screening for cancer and can control the risks of cardiovascular diseases [3, 12, 13].

Community health workers have been widely used in low- and middle-income countries to scale up HIV care. In rural Haiti, CHWs have served to bridge gaps in access to care which arise from lack of communication in terms of patient follow-up and long distances which patients travel to address health problems [14]. Such cadres have been involved in directly observed administration of tuberculosis medicines since the mid-1980s in Haiti [14]. A comparative assessment done on the integration of community home-based care programmes within national primary health care revitalisation strategies in Ethiopia, Malawi, South Africa and Zambia highlights some common strengths across the four countries. Community home-based care programmes in these countries, in which service delivery cuts across health and non-health domains and begins to extend beyond one priority disease, bring a vital resource to the primary health care revitalisation agenda. Furthermore, they support the establishment of primary care networks which are capable of addressing the health and social care demands of patients with chronic conditions [15]. South Africa has implemented CHW programmes for decades; however, the country failed to include them in the post-apartheid health system [16]. The CHW programme in South Africa dates back to as early as 1940, with the establishment of the Pholela Health Centre by two medical doctors, Sydney and Emily Kark [17].

The Centre’s task was to promote curative and preventive services [17]. During this era, the first five community health workers operated from the centre and each was responsible for 4000–5000 people. Communicable disease control and community health education were their main tasks [17]. The model being used for CHW (community health worker) programmes in South Africa has its roots in the Pholela Health Centre community-oriented primary health (COPC) model. Lately, South Africa is including CHWs as important members of community-based health programmes using Brazil’s model, in which CHWs form the foundation of the health care system.

In 2010, South Africa’s National Department of Health (NDoH) launched a national primary health care (PHC) initiative to strengthen health promotion, disease prevention and early disease detection. The strategy, called Re-engineering Primary Health Care, aims to support a preventive and health-promoting community-based PHC model by using community-based outreach teams (known in South Africa as ward-based primary health care outreach teams or WBPHCOTs). The WBPHCOTs are staffed by CHWs under the supervision of facility-based nurses. These teams provide health education, promote healthy behaviours, assess community health needs, manage minor health problems and support linkages to health services and health facilities [18]. The recommendation is that ward-based primary health care outreach teams should comprise a professional nurse who is the outreach team leader, a health promotion officer, an environmental health officer and six CHWs [19]. A gap remains between policy and implementation, especially in rural districts which needs to be addressed [20]. In South Africa, the WBPHCOTs have been established, registered and have been reporting their activities through the District Health Information System (DHIS) since January 2012; however, the concern is that WBPHCOTs are not functioning optimally, while still constituting a large part of the KwaZulu-Natal Department of Health’s budget. In this study, the researcher will explore roles and responsibilities of community health workers in WBPHCOTs. In addition, the aim of South Africa’s health care system is to strengthen health services and improve accessibility to the community through primary health care re-engineering. This programme comprises the nurses and the community health workers. Although the promise of WBPHCOTs is improving community health, several factors are undermining its success. Key among these challenges are as follows: varying perceptions of the CHW roles, lack of knowledge and skills and lack of stakeholders and community support [21–24].

### The roles and responsibilities of the community health workers

In South Africa, there has been a notable increase in the number of community health workers in the last decade [25]. Community health worker is an umbrella name used for all the lay health work in the health system in the context of South Africa health sector. This concept emerged in 1980s when the non-governmental organisations (NGOs) began to employ community-based carers at home [6, 26]. This concept was introduced and adopted in the policy framework for health sector by the government thus reducing the challenge of human resources in the health sector in South Africa. The importance of CHW in the health sector cannot be overemphasised in South Africa hence the need to explore the roles and responsibilities of CHW in the twenty-first century where there is increasing need for prevention of diseases and promoting health and wellness. CHW is not new in South Africa as well as globally due to the 1978 Alma Ata Declaration on primary health care which promote CHW initiatives in low- and middle-income countries.

The following are the roles and responsibilities of the CHW:

- They create a voice for the people
- They serve as a bridge between patient, communities and health system
- They act as lay counsellors
- They fulfil identity-related needs
- They run campaign programmes to mobilise communities’ members for health services
- They target households’ coverage for health care service [6, 26, 27]

Based on the above roles, it is essential to ascertain if CHWs are indeed adding value in the health system.

### Aim of the study

The aim of this scoping review is to describe the roles and responsibilities WBPHCOTs in KwaZulu-Natal.

### Research objective

To explore and describe the roles and responsibilities of community health workers who are members of ward-based primary health care outreach teams.

### Research question

What are the roles and responsibilities of ward-based primary health care outreach teams in KwaZulu-Natal?

## Methods

A scoping review of peer-reviewed and grey literature on the role and responsibilities of community health workers’ perceptions of ward-based primary health care outreach teams will be conducted. The review will include a quality assessment. This review will be guided by Arksey and O’Malley’s scoping review framework (Arksey and O’Malley, 2005) which stipulates the following steps:

1. Identifying the research question
2. Identifying relevant studies
3. Study selection
4. Charting the data
5. Collating, summarising and reporting the results

### Identifying the research question

The research question is: What are the roles and responsibilities of ward-based primary health care outreach teams in KwaZulu-Natal, are CHWs indeed adding value in the health system? Eligibility of research question: The study has used the Population, Concept and Context (PCC) according to the Joanna Briggs Institute Reviewers’ Manual, 2015 methodology framework to determine the eligibility of research question as illustrated in Table 1.

**Table 1:** PCC framework

Criteria	Determinants
Population	Community health worker
Concept	Ward-Based Primary Health Care Outreach Teams OR Primary Health care OR Community Home-based care OR Community healthcare OR Healthcare delivery in rural settings.
Context	Low- and middle-income countries, e.g. South Africa (KwaZulu-Natal)

### Identifying relevant studies

Primary studies with a clear empirical base utilising qualitative, quantitative and mixed methods published in peer-reviewed journals, as well as in grey literature, addressing the research question will be included. An electronic search of the following databases will be conducted: Embase, PubMed, Google Scholar, Science Direct, Clinical key and from the EBSCOhost platform and Dissertation via World Cat. In addition, articles will also be searched through the “cited by” search, as well as a search of citations included in the reference lists of articles included. Websites such as those of the World Health Organization (WHO) and government websites will be searched for policies and guidelines for ward-based primary health care outreach teams. The search terms will include the terms: community health worker, Primary Health Care, ward-based primary health care outreach teams. Duplicates will be removed, and the studies will be screened against the inclusion and exclusion criteria.

### Study selection

The eligibility criteria were developed to ensure that specific information relating to the research question is included in the studies.

### Inclusion criteria

For studies to be included, they will meet the following criteria:

- Be in all languages
- Be available in full text
- Include the terms community health work, primary health care and ward-based primary health care outreach teams
- Conducted in low- and middle-income countries

### Exclusion criteria

Studies will be excluded if they display any of the characteristics listed below:

- Studies do not include the terms primary health care and community health worker
- Reviewed articles
- Studies not available in full text
- Studies not related to population and concept of interest
- Reviews articles, letters to the editors and opinion pieces
- Studies not from low- and middle-income countries

### Search strategy

**Fig. 1:**
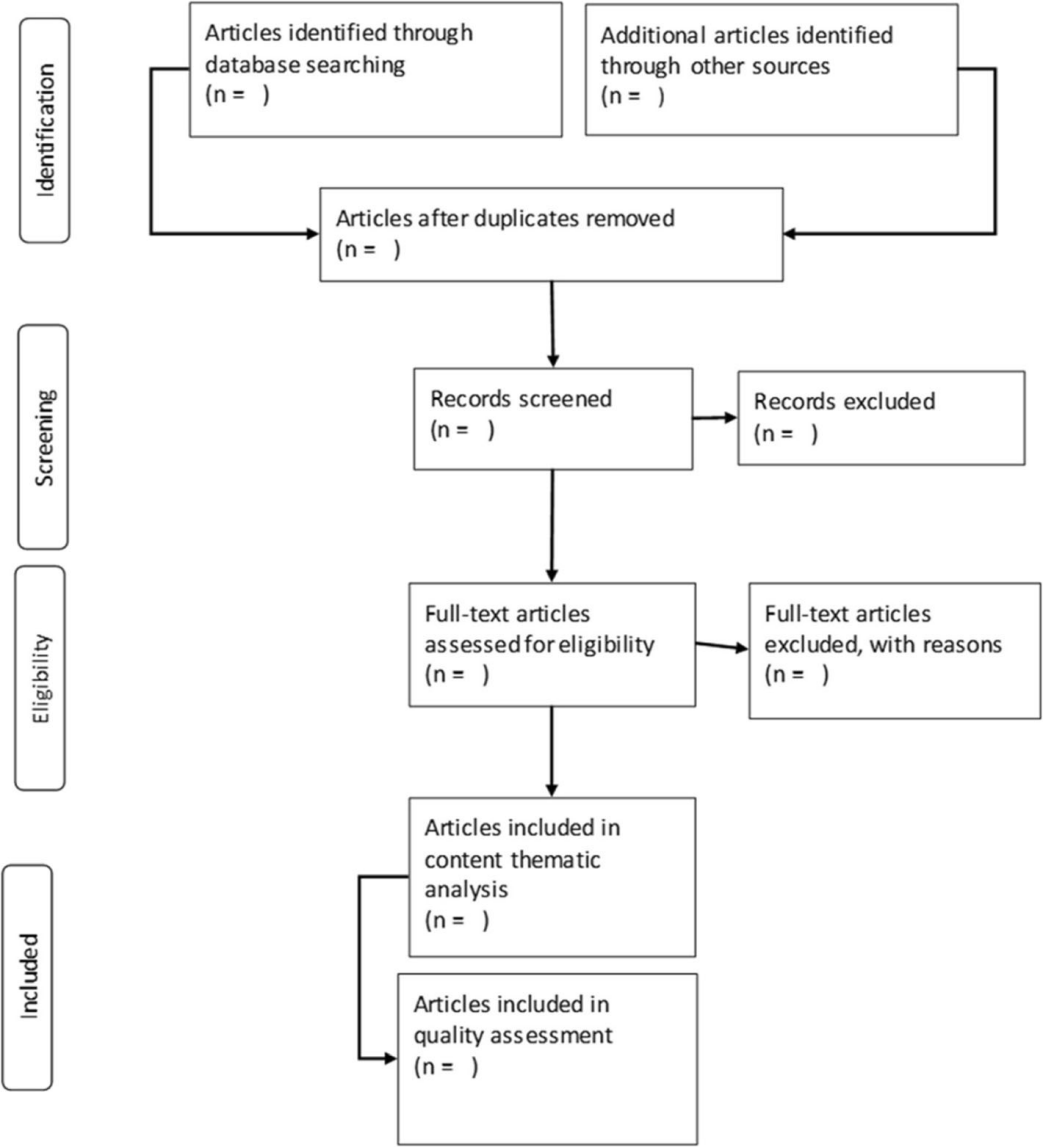
A PRISMA flowchart showing the process the search will follow from literature

The search strategy was piloted to check the appropriateness of the selected electronic databases and keywords (Table 2). A new Endnote library will be created for this review. The principal investigator will conduct a comprehensive search and screening of the study titles from the abovementioned databases. All studies with eligible titles will be exported to the endnote library, and all duplicates will be removed before abstract screening. Two reviewers will independently conduct abstract screening and full article screening of selected studies with guidance from the eligibility criteria. However, if there is any discrepancy, it will be resolved by reaching an agreement or a third reviewer will be introduced. The local school library services will be utilised in order to optimise the article search procedure. The UKZN library services will assist with retrieving and finding articles to be included in the full article screening. Authors will also be contacted for electronically unavailable papers. The screening results will be reported by use of the adapted PRISMA chart as in Fig. 1.

**Table 2:** Piloted database search results

Date of search	Keywords	Search engine	No. of publication retrieved	Search terms
07 May 2018	Community Health Worker AND Primary Health Care	PubMed	4003	(“community health workers”[MeSH Terms] OR (“community”[All Fields] AND “primary health care”[All Fields].

#### Charting of data

A data charting table will be used to extract background information and process the information from each study utilised. A data charting form, highlighting the important aspects for the study, will be developed and piloted. The variables and themes included will answer the research question. The structured data matrix form will be continually updated.

Table 3 represents the data charting.

**Table 3:** Data charting table

Author and date	
Countries	
Aims of the study	
Study design	
Study population	
Intervention	
Perception of CHW about WBPHCOTs	
Perceived responsibilities and role of CHW	
Quality appraisal	
Findings	
Conclusions/recommendation	

#### Collating, summarising and reporting the results

A narrative account of the data extracted from the included studies will be analysed using thematic content analysis. Data will be extracted around the following themes: perceptions, roles and responsibilities and community health care workers in ward-based primary health care outreach teams. Emerging themes will also be extracted. The NVIVO software will be used to code and analyse data from studies included.

#### Quality appraisal

The Mixed Method Appraisal Tool (MMAT) version 2011 [28] will be used to determine the methodological quality of the studies that will be included in our search. The advantage of using the MMAT for this review is that it allows reviewers to assess the methodological quality of all the qualitative, quantitative and mixed methods research studies that will be included in the scoping review. The tool will be used to examine the appropriateness of the aim of the study, the adequacy and methodology, the study design, data collection, study selection, data analysis, presentation of findings and the authors’ discussions and conclusions in order to determine the quality of article.

## Discussion

This scoping review will evaluate the documented evidence that suggests the roles and responsibilities of ward-based primary health care outreach teams. The review will also highlight factors enabling or hampering the effectiveness of ward-based primary health care outreach teams. The rigorous and systematic nature of this review will ensure that it captures all relevant information on ward-based primary health care outreach teams. This review will explore areas where the WBPHCOTs are not functioning well and make recommendations for improvement, as well as identify areas where they are functioning well and suggest ways of replicating these. Results from this scoping review will be useful to inform programme implementers of the impact made by these teams on health and health care in the country.

## Data Availability

The data and the material will be made available on request.

## Abbreviations

CHW: Community health worker
MMAT: Mixed Method Appraisal Tool
PCC: Population, Concept and Context
PHC: Primary health care
PRISMA: Preferred Reporting Items for Systematic Reviews and Meta-Analyses
WBPHCOTs: Ward-based primary health care outreach teams
